# Identification of the Genomic Region Underlying Seed Weight per Plant in Soybean (*Glycine max* L. Merr.) via High-Throughput Single-Nucleotide Polymorphisms and a Genome-Wide Association Study

**DOI:** 10.3389/fpls.2018.01392

**Published:** 2018-10-11

**Authors:** Yan Jing, Xue Zhao, Jinyang Wang, Weili Teng, Lijuan Qiu, Yingpeng Han, Wenbin Li

**Affiliations:** ^1^Key Laboratory of Soybean Biology in Chinese Ministry of Education (Key Laboratory of Soybean Biology and Breeding/Genetics of Chinese Agriculture Ministry), Northeast Agricultural University, Harbin, China; ^2^National Key Facility for Crop Gene Resources and Genetic Improvement, Institute of Crop Science, Chinese Academy of Agricultural Sciences, Beijing, China

**Keywords:** seed weight per plant, soybean, single nucleotide polymorphism, genome-wide association analysis, candidate genes

## Abstract

Seed weight per plant (SWPP) of soybean (*Glycine max* (L.) Merr.), a complicated quantitative trait controlled by multiple genes, was positively associated with soybean seed yields. In the present study, a natural soybean population containing 185 diverse accessions primarily from China was used to analyze the genetic basis of SWPP via genome-wide association analysis (GWAS) based on high-throughput single-nucleotide polymorphisms (SNPs) generated by the Specific Locus Amplified Fragment Sequencing (SLAF-seq) method. A total of 33,149 SNPs were finally identified with minor allele frequencies (MAF) > 5% which were present in 97% of all the genotypes. Twenty association signals associated with SWPP were detected via GWAS. Among these signals, eight SNPs were novel loci, and the other twelve SNPs were overlapped or located in the linked genomic regions of the reported QTL from SoyBase database. Several genes belonging to the categories of hormone pathways, RNA regulation of transcription in plant development, ubiquitin, transporting systems, and other metabolisms were considered as candidate genes associated with SWPP. Furthermore, nine genes from the flanking region of Gm07:19488264, Gm08:15768591, Gm08:15768603, or Gm18:23052511 were significantly associated with SWPP and were stable among multiple environments. Nine out of 18 haplotypes from nine genes showed the effect of increasing SWPP. The identified loci along with the beneficial alleles and candidate genes could be of great value for studying the molecular mechanisms underlying SWPP and for improving the potential seed yield of soybean in the future.

## Introduction

Seed weight per plant, a complicated and agronomically important quantitative trait, was significantly related with yield in soybean (*Glycine max* L. Merr.) (Chen et al., 2007; Liu et al., 2016). SWPP is controlled by multiple genes or quantitative trait loci (QTL). An additive effect dominates the inheritance pattern of SWPP (Chen et al., 2007). The development of cultivars with suitable SWPP has been an important breeding object of many soybean breeders because SWPP was an important soybean yield component. SWPP is influenced by the environment or genotype by environment interactions, and this trait performs differently in different environments. Hence, breeding soybean cultivars with suitable SWPP via traditional methods requires evaluation in multiple environments over several years, which is expensive, time consuming, and labor-intensive.

The advances in molecular marker technologies have enabled the efficient elucidation of the genetic architecture of soybean SWPP. To date, fewer than 50 QTL, located on chromosome (Chr.) 3 (linkage group, LG N), Chr.4 (LG C1), Chr.5 (LG A1), Chr.6 (LG C2), Chr.7 (LG M), Chr.8 (LG A2), Chr.9 (LG K), Chr.10 (LG O), Chr.18 (LG G), and Chr.19 (LG L), have been reported in the SoyBase databank^1^. Of these identified QTL, some QTL, located on Chr. 3 (LG N), Chr.4 (LG C1), and Chr.6 (LG C2), were verified by many studies (Chen et al., 2007; Kuroda et al., 2013; Yao et al., 2015). The genetic maps used in most of these studies had incomplete coverage of the soybean genome, with large gaps in the maps. Thus, some QTL were difficult to directly apply for marker assisted selection (MAS) of SWPP.

Genome-wide association studies (GWAS), based on high-density markers and natural populations, have been regarded as an efficient alternative to linkage analysis with more extensive recombination events and shorter LD segments. Thus, the resolution and accuracy of marker-phenotype associations could be further increased via GWAS compared with the conventional QTL mapping of segregating populations (Li et al., 2015). To date, GWAS has been widely utilized to elucidate the genetic basis of many complex traits in some crops, including rice (Huang et al., 2010), maize (Weng et al., 2011), wheat (Raman et al., 2010), and barley (Cockram et al., 2010). In soybean, the genetic architecture of some important traits, such as protein (Hwang et al., 2014), fatty acid content (Li et al., 2015), and SCN resistance (Han et al., 2015; Zhao et al., 2017), has been well dissected. Moreover, the rapid development of next-generation sequencing technology and SNP genotyping technology has propelled much of the practicability of GWAS. In previous studies, Yan et al. (2017), Contreras-Soto et al. (2017), and Zhang et al. (2016) identified seventeen, seven, and forty-eight SNPs, respectively, which were all significantly associated with 100-seed weight of soybean (HSW). However, to date, only few studies were conducted to identify QTL underlying SWPP based on high-throughput sequencing technology. Han et al. (2016) detected rs18976374 (located on Chr.16) significantly related with SWPP by using a bar coded multiplex sequencing approach with an Illumina Genome Analyzer II. Liu et al. (2016) found one SNP (ss244932137 located on Chr.3) associated with SWPP through GWAS strategy based on an association panel of 138 cultivars genotyped by Illumina SoySNP6KiSelect BeadChip.

The aims of the present study were to understand better the genetic architecture of SWPP via GWAS based on 185 tested accessions and 33,149 SNPs and to analyze the potential candidate genes that might regulate soybean SWPP in associated genomic regions with peak SNPs.

## Materials and Methods

### Soybean Germplasms and Field Trials

A total of 185 diverse soybean accessions (**Supplementary Table S1**), including landraces and elite cultivars, were applied to evaluate the variation of SWPP and for the subsequent sequencing analysis. All samples were grown in Harbin in 2015 and 2016, Gongzhuling in 2015 and 2016, and Shenyang in 2015 and 2016. Field experiments were performed with single row plots of 3 m in length with 0.65 m between rows. A randomized complete block design was used with three replications in each tested environment. After reaching full maturity, a total of 10 randomly selected plants per row in each plot were weighed and used to evaluate SWPP.

### DNA Isolation and Sequencing Analysis

Total DNA from the fresh leaves of a single test sample was extracted by the CTAB method according to Han et al. (2015). The isolated high-quality DNA was partly sequenced via specific locus amplified fragment sequencing (SLAF-seq) methodology (Sun et al., 2013). Soybean reference genome (Version:Glyma.Wm82.a2) was preliminarily analyzed and digested enzyme *Mse I* (EC 3.1.21.4) and *Hae III* (EC: 3.1.21.4) (Thermo Fisher Scientific Inc., Waltham, MA, United States) were used to generate more than 50,000 sequencing tags (approximately 300–500 bp in length) of all tested samples. The obtained tags were evenly distributed among the unique genomic regions of the 20 soybean chromosomes. The sequencing libraries of each tested accession were built based on the sequencing tags. A barcode method combined with the Illumina Genome Analyzer II system (Illumina Inc., San Diego, CA, United States) was used to generate the 45-bp sequence reads at both ends of the sequencing tags from each accession library. Short Oligonucleotide Alignment Program 2 (SOAP2) software was used to align raw paired-end reads to the soybean reference genome (Version:Glyma.Wm82.a2). The SLAF groups were designed based on the raw reads, which mapped to the same unique genomic positions. Approximately 58,000 high-quality SLAF tags were acquired from each tested accession. The SNPs were called as such based on MAF ≥ 0.05. If the minor allele depth or the total depth of the sample was larger than 1/3, then the genotype was considered heterozygous.

For thirty lines, a genome resequencing with 10-fold in depth was conducted on an Illumina HiSeq 2000 sequencer. Paired-end resequencing reads were mapped to the soybean Williams 82 reference genome (Version:Glyma.Wm82.a2) with BWA (Version: 0.6.1-r104) (Zhou et al., 2015) using the default parameters. SAMtools48 (Version: 0.1.18) software (Zhou et al., 2015) was used for converting mapping results into the BAM format and to filter the unmapped and non-unique reads. Duplicated reads were filtered with the Picard package (picard.sourceforge.net, Version:1.87) (Zhou et al., 2015). The BEDtools (Version: 2.17.0) (Zhou et al., 2015) coverageBed program was applied to compute the coverage of sequence alignments. A sequence was defined as absent when the coverage was lower than 90% and present when the coverage was higher than 90%. SNP detection was performed by the Genome Analysis Toolkit (GATK, version 2.4-7-g5e89f01) and SAMtools (Zhou et al., 2015). Only the SNPs detected by both methods could be analyzed further. SNPs with allele frequencies lower than 1% in the population were discarded. SNP annotation was performed based on the soybean genome (Version:Glyma.Wm82.a2) using the package ANNOVAR (Version: 2013-08-23) (Zhou et al., 2015).

### Population Structure Evaluation and Linkage Disequilibrium (LD) Analysis

The population structure analysis of the natural group was conducted based on the PCA programs in the GAPIT software package (Lipka et al., 2012). The LD block was evaluated across the soybean genome based on SNPs with MAF ≥ 0.05 and missing data ≤ 10% by using squared allele frequency correlations (r^2^) in TASSEL version 3.0 (Bradbury et al., 2007). In contrast to the GWAS, missing SNP genotypes were not imputed with the major allele prior to LD analysis. The parameters in the software programs were set according to the MAF (≥0.05) and the integrity of each SNP (≥80%).

### Genome-Wide Association Analysis

The SWPP association signals were identified based on 33,149 SNPs (**Supplementary Table S2**) from 185 tested samples with CMLM in GAPIT (Lipka et al., 2012). The *P* value was calculated using the Bonferroni method with α ≤ 0.05 (≤2.58 × 10^-4^) and was used as the threshold to declare whether a significant association signal existed (Holm, 1979).

### Prediction of Candidate Genes Controlling SWPP

According to the studies of Hwang et al. (2014), Han et al. (2015), and Zhao et al. (2017), the average LD decay distance of soybean genome (Version:Glyma.Wm82.a2) was approximately 200 kbp. Thus, candidate genes located in the 200-kbp genomic region of each peak SNP were classified and annotated underlying the soybean reference genome Williams 82^2^.

### Haplotype Analysis of Candidate Genes

Based on the genome annotation, SNPs were classified in exonic regions (overlapping with a coding exon), splicing sites (within 2 bp of a splicing junction), 5′UTRs and 3′UTRs, intronic regions (overlapping with an intron), upstream and downstream regions (within a 1 kb region upstream or downstream from the transcription start site), and intergenic regions. SNPs in coding exons were further grouped into synonymous SNPs (did not cause amino acid changes) and nonsynonymous SNPs (caused amino acid changes). The variation happened in these regions (except for intergenic regions) of candidate genes in thirty lines generated from genome re-sequencing data which were analyzed using the General Linear Model (GLM) method in TASSEL version 3.0 (Bradbury et al., 2007) to identify related SNPs and haplotypes. Significant SNPs affecting the SWPP were claimed when the test statistics reached *P* < 0.01.

## Results

### Statistical and Variation Analysis of SWPP

**FIGURE 1:**
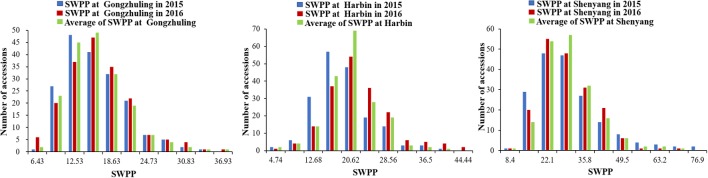
Distribution of seed weight per plant (SWPP) among 185 soybean accessions.

The SWPP of the 185 tested soybean accessions, grown in multiple locations over years, was determined. The mean results of the SWPP analysis showed that the effects of genotype, environment, and genotype by environment interactions were significant. The skewness and kurtosis of SWPP across the six environments were both less than 1, indicating continuous variation and a near normal distribution (**Figure 1**). Therefore, the SWPP in the present study was suitable for the subsequent GWAS.

### Specific-Locus Amplified Fragment Sequencing (SLAF-seq) and Genotyping

**FIGURE 2:**
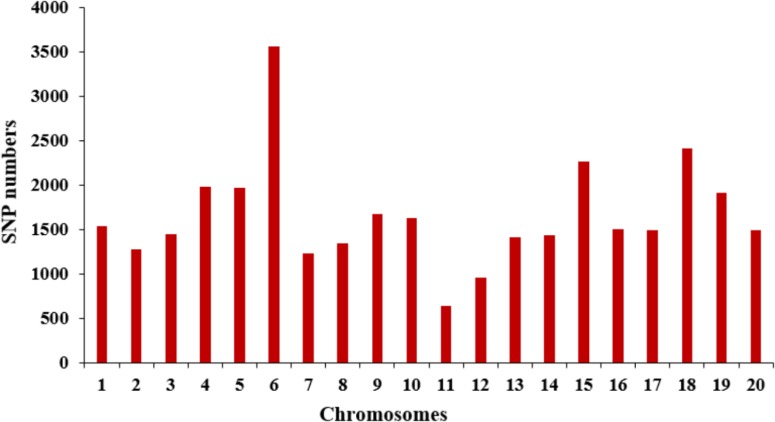
Distribution of SNP markers across 20 soybean chromosomes.

The selective population contained 185 diverse accessions, primarily collected from China. The genomic DNA from all tested accessions was extracted and partially sequenced based on the SLAF-seq approach. For each tested sample, a mean of 49,571 high-quality tags (or SLAFs) was scanned from 153 million paired-end reads with a 45-bp read length and 6.51-fold sequencing depth. A total of 33,149 SNPs with MAF ≥ 0.05 were generated from the high-quality tags and subsequently used to perform GWAS for SWPP. The obtained SNPs were evenly distributed among the 20 soybean chromosomes, resulting in a marker density of 28.7 kbp per SNP. Chr. 6 and Chr. 11 included the most and least numbers of SNPs, which was 3,556 and 638, respectively (**Figure 2**).

### Extent of Linkage Disequilibrium (LD) and Analysis of Population Structure

**FIGURE 3:**
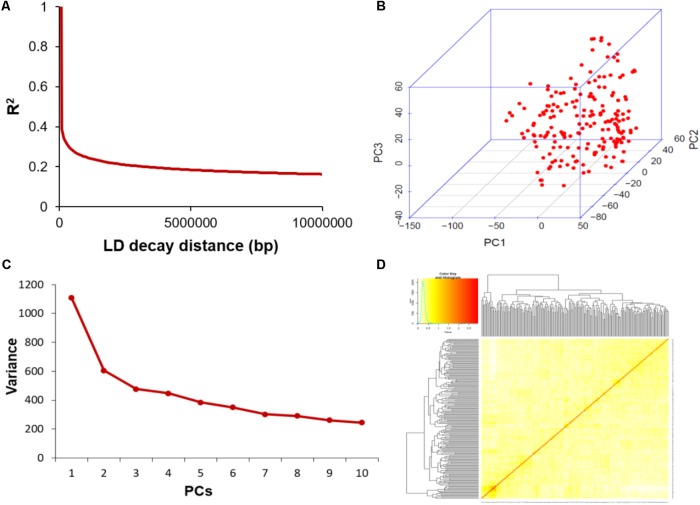
Linkage disequilibrium (LD), principal component, and kinship analyses of soybean genetic data. **(A)** LD decay of the genome-wide association study (GWAS) population. **(B)** The first three principal components of more than 30,000 SNPs used in the GWAS. **(C)** Population structure of soybean germplasm collection reflected by principal components. **(D)** A heatmap of the kinship matrix of the 185 soybean accessions calculated from the same SNPs.

The average distance of LD decay was analyzed to characterize the mapping resolution for GWAS, and LD decayed differently among all the 20 chromosomes. Accordingly, the mean LD decay of the panel was evaluated at 214 kbp when r^2^ dropped to half of its maximum value (**Figure 3A**). To scan the population stratification of the association panel, principal component analysis and kinship analysis were conducted based on all 33,149 SNP markers. The first three PCs explained 13.83% of the genetic variation. A drastic decline in the genetic variation appeared at PC2 (**Figure 3B**). However, analysis of the variation of the first 10 PCs revealed an inflection point at PC3 (**Figure 3C**). Thus, these results suggested that mainly the first three PCs dominated the population structure. Additionally, the heatmap of kinship matrix revealed low levels of genetic relatedness among the 185 tested samples (**Figure 3D**).

### Quantitative Trait Nucleotide (QTN) Associated With SWPP Evaluated by GWAS

**FIGURE 4:**
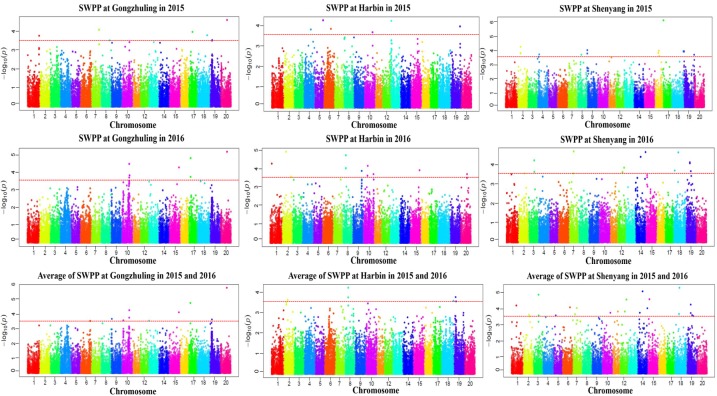
Manhattan plot of association mapping of the SWPP in soybean.

A total of 20 association signals, distributed on 12 of the 20 soybean chromosomes, were detected by CMLM in the present study (**Figure 4** and **Table 1**). Among these signals, only one QTN (Gm18:23052511 located on Chr.18) was identified in the linked region of a known SWPP QTL. However, as a specific member of seed weight, another 11 SNPs were overlapped or located in the linked region of a known seed weight QTL from the SoyBase databank, particularly for 100-seed weight (**Table 2**). The remaining 8 association signals were regarded as novel loci, which were first reported for SWPP in the present study (**Table 2**). The average SWPP for all tested samples with two different alleles were compared (**Table 1**), and the results demonstrated that the SWPP among these samples were so different that the appropriate alleles might be effectively applied in the marker assisted selection (MAS) of soybean cultivars with suitable SWPP.

**Table 1:** Peak SNP associated with SWPP and the evaluation of beneficial alleles.

SNP^a^	Chr.^b^	Position	Location	Year	-log10(P)	MAF^c^	Allele 1	Allele 2	Average SWPP of accessions with allele 1^d^	Average SWPP of accessions with allele 2^d^	Average SWPP of population^d^
Gm02:17792241	2	17792241	Harbin	2016	4.91	0.05	G	T	27.33	19.71	20.07
			Harbin	Average	3.63				27.53	18.66	19.01
Gm02:27731352	2	27731352	Shenyang	2016	3.55	0.05	C	A	32.57	25.37	25.91
			Shenyang	Average	3.64				30.4	24.95	25.48
Gm03:25789435	3	25789435	Shenyang	2016	4.22	0.06	A	G	31.14	25.73	25.91
			Shenyang	Average	4.85				31.81	25.28	25.48
Gm03:26388034	3	26388034	Shenyang	2016	3.63	0.05	G	T	26.20	19.19	25.91
			Shenyang	Average	3.58				25.73	19.83	25.48
Gm07:19488264	7	19488264	Shenyang	2016	3.55	0.06	G	T	28.00	25.84	25.91
			Shenyang	Average	4.04				27.1	25.34	25.48
Gm08:15768591	8	15768591	Harbin	2016	4.72	0.22	T	C	23.07	19.15	20.07
			Harbin	Average	4.22				21.75	18.19	19.01
Gm08:15768603	8	15768603	Harbin	2016	4.04	0.27	A	G	21.76	19.42	20.07
			Harbin	Average	3.75				20.62	18.39	19.01
Gm10:34747895	10	34747895	Gongzhuling	2016	3.70	0.07	T	C	20.10	19.95	20.04
			Gongzhuling	Average	3.76				19.64	18.9	19.58
Gm10:34946492	10	34946492	Gongzhuling	2016	4.47	0.05	C	T	20.07	19.68	20.04
			Gongzhuling	Average	4.25				19.65	18.22	19.58
Gm12:24101071	12	24101071	Shenyang	2016	3.61	0.06	C	A	30.35	25.83	25.91
			Shenyang	Average	3.82				30.47	25.38	25.48
Gm12:31782089	12	31782089	Shenyang	2016	3.84	0.05	C	A	28.76	25.76	25.91
			Shenyang	Average	4.57				27.71	25.45	25.48
Gm14:24306475	14	24306475	Shenyang	2016	4.41	0.05	G	T	26.40	25.88	25.91
			Shenyang	Average	5.07				25.55	25.39	25.48
Gm14:47326193	14	47326193	Shenyang	2016	4.64	0.06	T	G	25.93	23.37	25.91
			Shenyang	Average	4.03				25.63	22.96	25.48
Gm15:45663581	15	45663581	Gongzhuling	2016	4.29	0.07	T	A	26.24	19.86	20.04
			Gongzhuling	Average	4.11				24.28	19.46	19.58
Gm17:10196755	17	10196755	Gongzhuling	2016	4.82	0.05	A	C	20.91	19.97	20.04
			Gongzhuling	Average	4.74				19.68	19.52	19.58
Gm18:23052511	18	23052511	Shenyang	2016	4.63	0.05	C	A	25.97	19.97	25.91
			Shenyang	Average	5.29				25.53	20.37	25.48
Gm19:11082560	19	11082560	Gongzhuling	2015	3.53	0.41	C	T	19.88	19.27	19.58
			Gongzhuling	Average	3.62				19.67	19.51	19.58
Gm19:19848537	19	19848537	Shenyang	2016	4.07	0.06	G	A	31.79	25.53	25.91
			Shenyang	Average	3.73				28.96	25.26	25.48
Gm19:19848932	19	19848932	Shenyang	2016	4.13	0.06	G	A	32.46	25.49	25.91
			Shenyang	Average	4.25				28.83	25.26	25.48
Gm20:29716176	20	29716176	Gongzhuling	2015	4.61	0.05	A	C	19.63	19.18	19.58
			Gongzhuling	2016	5.17				20.11	19.36	20.04
			Gongzhuling	Average	5.77				19.61	19.38	19.58


*^*a*^single nucleotide polymorphisms; ^*b*^chromosome; ^*c*^minor allele frequency; ^*d*^gram.*

**Table 2:** Significant SNPs and candidate genes associated with SWPP.

SNP^a^	Chr.^b^	Position	Known QTL	References	Gene	Distance to SNP(Kbp)	Functional annotations
Gm02:17792241	2	17792241	Seed weight 34-3	Han et al., 2012	Glyma.02G159500	45.00	C2H2 and C2HC zinc fingers superfamily protein
					Glyma.02G159600	41.97	hAT transposon superfamily
					Glyma.02G159900	2.34	pleiotropic drug resistance 11
					Glyma.02G160000	17.07	zinc ion binding; nucleic acid binding
Gm02:27731352	2	27731352			Glyma.02G173900	29.78	KH domain-containing protein/zinc finger (CCCH type) family protein
					Glyma.02G174100	54.94	SNF2 domain-containing protein/helicase domain-containing protein
					Glyma.02G174200	74.67	Nodulin MtN21/EamA-like transporter family protein
Gm03:25789435	3	25789435			Glyma.03G086900	51.99	GDSL-like Lipase/Acylhydrolase superfamily protein
Gm03:26388034	3	26388034			Glyma.03G088500	85.93	RNA polymerase II, Rpb4, core protein
					Glyma.03G088800	58.73	AGC kinase family protein
					Glyma.03G088900	85.80	auxin-responsive family protein
Gm07:19488264	7	19488264	Seed weight 12-4	Csanadi et al., 2001	Glyma.07G157500	67.24	hAT dimerization domain-containing protein / transposase-related
					Glyma.07G157600	25.92	Nucleic acid-binding, OB-fold-like protein
					Glyma.07G157700	14.16	PIF1 helicase
					Glyma.07G157800	0.02	Cyclophilin-like peptidyl prolyl *cis-trans* isomerase family protein
					Glyma.07G157900	27.01	SNF2 domain-containing protein / helicase domain-containing protein
Gm08:15768591	8	15768591	Seed weight	Zhang et al., 2004/Han et al., 2012	Glyma.08G194600	87.34	Multidrug resistance associated protein 6
Gm08:15768603		15768603	22-1/Seed weight 35-1				
					Glyma.08G194900	63.78	Pyridoxal phosphate phosphatase related protein
					Glyma.08G195200	40.42	Protein phosphatase 2A, regulatory subunit PR55
					Glyma.08G195300	22.24	DOF zinc finger protein 1
					Glyma.08G195400	17.31	F-box/RNI-like/FBD-like domains containing protein
					Glyma.08G195500	10.81	C2H2 and C2HC zinc fingers superfamily protein
					Glyma.08G195600	9.55	F-box/RNI-like superfamily protein
					Glyma.08G195700	2.34	C2H2 and C2HC zinc fingers superfamily protein
					Glyma.08G195800	9.82	C2H2 and C2HC zinc fingers superfamily protein
					Glyma.08G195900	15.42	Ubiquitin-specific protease 6
					Glyma.08G196100	34.08	Dentin sialophosphoprotein-related
					Glyma.08G196200	45.51	Amino acid dehydrogenase family protein
					Glyma.08G196300	56.32	Ubiquitin conjugating enzyme family protein
					Glyma.08G196400	71.62	Tudor / PWWP / MBT superfamily protein
					Glyma.08G196500	80.14	Ribosomal L28e protein family
Gm10:34747895	10	34747895	Seed weight 34-8	Han et al., 2012	Glyma.10G129300	92.46	Ubiquitin C-terminal hydrolase 3
Gm10:34946492	10	34946492	Seed weight 34-8	Han et al., 2012	Glyma.10G129700	85.64	Disease resistance family protein / LRR family protein


					Glyma.10G129800	34.31	Myosin family protein with Dil domain
					Glyma.10G130000	44.84	Aluminum-induced protein with YGL and LRDR motifs
					Glyma.10G130100	57.1	Calcium-dependent lipid binding (CaLB domain) family protein
Gm12:24101071	12	24101071	Seed weight	Han et al., 2012/Kato et al., 2014	Glyma.12G154400	85.95	Cytochrome c oxidase 17
Gm12:31782089	12	31782089	35-4/Seed weight 50-15				
					Glyma.12G163800	48.65	Major facilitator superfamily protein
					Glyma.12G164100	80.92	Auxin response factor 1
					Glyma.12G164200	92.20	EMBRYO DEFECTIVE 140
Gm14:24306475	14	24306475	Seed weight	Hoeck et al., 2003	Glyma.14G136800	59.90	ATP phosphoribosyl transferase 2
Gm14:47326193	14	47326193	13-2				
					Glyma.14G136900	71.77	Cellulose-synthase-like C5
					Glyma.14G207000	61.45	Yippee family putative zinc-binding protein
					Glyma.14G207100	59.98	Tetratricopeptide repeat (TPR)-like superfamily protein
					Glyma.14G207200	35.3	ABC transporter of the mitochondrion 3
					Glyma.14G207300	13.48	Tetratricopeptide repeat (TPR)-like superfamily protein
					Glyma.14G207500	5.30	TRAM, LAG1 and CLN8 (TLC) lipid-sensing domain containing protein
					Glyma.14G207600	11.92	Cystathionine beta-synthase (CBS) family protein
					Glyma.14G207700	39.72	Thiamine pyrophosphate dependent pyruvate decarboxylase family protein
					Glyma.14G208000	52.97	Late embryogenesis abundant (LEA) hydroxyproline-rich glycoprotein family
					Glyma.14G208200	65.81	AMP-dependent synthetase and ligase family protein
					Glyma.14G208400	77.79	NC domain containing protein related
					Glyma.14G208500	86.61	Auxin response factor 8
Gm15:45663581	15	45663581			Glyma.15G240600	31.54	Senescence-related gene 1
					Glyma.15G240700	18.30	MAP kinase 19
					Glyma.15G240800	51.16	pfkbp-like carbohydrate kinase family protein
					Glyma.15G241000	79.81	Multidrug resistance-associated protein 6
Gm17:10196755	17	10196755	Seed weight 13-5/Seed weight 49-10	Hoeck et al., 2003/Teng et al., 2009	Glyma.17G126800	74.02	Zinc finger C-x8-C-x5-C-x3-H type family protein
					Glyma.17G126900	67.85	RPM1-interacting protein 4 (RIN4) family protein
					Glyma.17G127000	56.77	GDSL-like Lipase / Acylhydrolase superfamily protein
					Glyma.17G127100	39.83	26S proteasome, regulatory subunit Rpn7; Proteasome component (PCI) domain
					Glyma.17G127200	35.30	Protein kinase superfamily protein
					Glyma.17G127400	24.28	Nucleoside diphosphate kinase 2
					Glyma.17G127700	8.92	Putative endonuclease or glycosyl hydrolase
					Glyma.17G127800	14.89	Lysine histidine transporter 1
					Glyma.17G128000	37.93	Malate synthase
					Glyma.17G128100	47.88	Malate synthase
					Glyma.17G128200	54.33	Protein kinase superfamily protein
					Glyma.17G128300	59.55	Adenylate kinase 1
					Glyma.17G128400	70.67	Homolog of nucleolar protein NOP56
					Glyma.17G128500	76.83	RNA-metabolizing metallo beta lactamase family protein
					Glyma.17G128600	93.68	Arginosuccinate synthase family
					Glyma.17G128700	99.79	Gamma-irradiation and mitomycin c induced 1
Gm18:23052511	18	23052511	Seed weight per plant 6-6	Yao et al., 2015	Glyma.18G143700	10.62	MATE efflux family protein
Gm19:11082560	19	11082560	Seed weight 13-10	Hoeck et al., 2003; Sebolt et al., 2000/Han et al., 2012	Glyma.19G057700	17.42	Germin-like protein 2
Gm19:19848537	19	19848537	Seed weight 9-1/Seed weight 34-5		Glyma.19G057800	69.03	BTB-POZ and MATH domain 6
Gm19:19848932	19	19848932			Glyma.19G067900	24.55	ABA-responsive element binding protein 3
Gm20:29716176	20	29716176			Glyma.20G079900	61.09	Coenzyme F420 hydrogenase family / dehydrogenase, beta subunit family


*^*a*^single nucleotide polymorphisms; ^*b*^chromosome*

### Prediction of Candidate Genes Controlling SWPP

The genes located in the 200-kbp genomic region of each peak SNP of the identified loci were considered as candidate genes, consistent with a mean LD decay distance of 214 kbp for the entire association panel. Approximately 126 candidate genes were identified. Among these genes, 26 genes had no functional annotation, and one gene had unknown function domains. The remaining 99 genes were classified into different groups by MAPMAN (Thimm et al., 2004) to determine clearly the potential functions. A total of 16 categories were scanned, including co-factor and vitamin metabolism, misc, RNA regulation of transcription, DNA synthesis/chromatin structure, protein synthesis/modification/degradation, signaling, development, transport, amino acid metabolism, hormone metabolism, abiotic stress, cell wall, major CHO metabolism, nucleotide metabolism, other groups, and unassigned genes (**Supplementary Figure S1**).

The factors that influenced SWPP were almost the same as those that influence seed weight, and seed size was the main component for determining seed weight. Thus, the factors that could regulate the mechanism of seed size might be important elements in directly or indirectly adjusting SWPP (Wang et al., 2017). In some plants, several genes controlling seed size/weight have been identified, including the genes associated with hormone metabolism, such as auxin and cytokinin (Schruff et al., 2006; Li et al., 2013), various transcription factors associated with RNA regulation of transcription in plant development and maturation, such as *TTG2, AP2*, *MINI3*, and *C2H2* (Garcia et al., 2005; Ohto et al., 2009; Costa et al., 2010; Yin et al., 2010; Cao et al., 2016), the genes associated with protein modification or degradation (particularly ubiquitin ligase genes) (Li et al., 2008; Xia et al., 2013; Wang et al., 2017), and regulatory genes dominating transport systems and other metabolic processes associated with plant growth and development, such as ABC transporters and amino acid metabolism (Wu et al., 2007; Kim et al., 2010; Less et al., 2010; Hildebrandt et al., 2015). Among these genes detected by GWAS in the present study, those associated with the RNA regulation of transcription, including different transcription factor families, such as *AP2* and *C2H2*, were scanned and identified as the functional genes for SWPP, including *Glyma.02G159600*, *Glyma.02G173900*, *Glyma.07G157600*, *Glyma.07G157800*, *Glyma.07G157900*, *Glyma.08G195300*, *Glyma.08G195500*, *Glyma.08G195600*, *Glyma.08G195700*, *Glyma.08G195800*, *Glyma.17G127400*, *Glyma.17G127700*, *Glyma.19G057700*, and *Glyma.19G067900* located on Chr.2, Chr.7, Chr.8, Chr.17, and Chr.19. Two brassinosteroid response factor genes (*Glyma.10G129700* and *Glyma.17G127100* with distances of 34.31 and 39.83 kbp from peak SNPs Gm10:34747895 and Gm17:10196755, respectively) were identified as candidate genes. Similarly, *Glyma.10G130000*, an auxin response factor gene located 44.84 kbp from SNP Gm10:34946492 on Chr.10, *Glyma.15G240600*, an ethylene factor gene located 31.54 kbp from SNP Gm15:45663581 on Chr.15, and *Glyma.17G127500*, a gibberellin factor gene located 15.58 kbp from SNP Gm17:10196755 on Chr.17, were all considered as the genes controlling SWPP. Three E3 ubiquitin ligase genes (*Glyma.08G195200*, *Glyma.08G195400*, and *Glyma.08G195900* with distances of 40.42, 17.31, and 15.42 kbp, respectively, from the peak SNP Gm08:15768591 located on Chr.8), which are associated with protein modification or degradation, might also affect SWPP. An additional 10 genes belonging to transport systems (such as ABC transporters) and other main metabolic processes (such as amino acid metabolism, N-metabolism, and vitamin metabolism), including *Glyma.02G159900*, *Glyma.08G196200*, *Glyma.12G163700*, *Glyma.12G163800*, *Glyma.14G207200*, *Glyma.14G207300*, *Glyma.14G207700*, *Glyma.14G207800*, *Glyma.17G127800*, and *Glyma.18G143700*, were also selected and might also contribute to SWPP.

**FIGURE 5:**
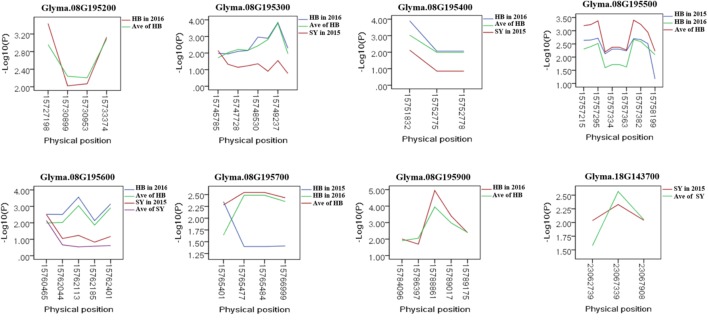
Candidate gene-based association. Gene-based association analysis of candidate genes with SNPs that were significantly correlated to SWPP. HB, Harbin; SY, Shenyang; Ave, Average seed weight per plant in 2015 and 2016.

**FIGURE 6:**
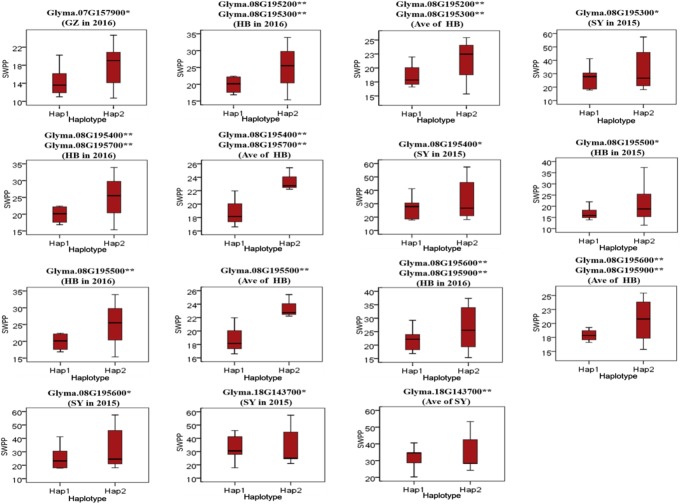
Haplotypes analysis of genes that related to SWPP. The ^∗^ and ^∗∗^ suggested significance of ANOVA at *p* < 0.05 and *p* < 0.01, respectively. HB, Harbin; GZ, Gongzhuling; SY, Shenyang; Ave, Average seed weight per plant in 2015 and 2016.

To predict the possible roles of candidate genes associated with SWPP, haplotype analysis of the 99 genes was performed. A total of 578 SNPs in 99 candidate genes were found among the thirty soybean lines (MAF > 0.1) through genome re-sequencing. Finally, 44 SNPs from nine genes were significantly associated with SWPP among multiple environments (**Figure 5** and **Supplementary Table S3**). *Glyma.07G157900*, with only one SNP, was not shown in the figure (**Figure 5**). Two haplotypes were identified for each of the nine genes and the SWPP between each pair of haplotypes showed significant or highly significant difference (**Figure 6**). *Glyma.07G157900*, from Gm07:19488264, was detected under the environments of Shenyang in 2016 and the average at Shenyang in 2015 and 2016, which was located in the genomic region of the known loci, “seed weight 12-4” (Csanadi et al., 2001). A total of seven genes from Gm08:15768591 and Gm08:15768603 were detected, which overlapped the region of the known loci, “seed weight 22-1” (Zhang et al., 2004) and “seed weight 35-1” (Han et al., 2012). Of the seven genes, *Glyma.08G195200* and *Glyma.08G195900* were detected under the environments of Harbin in 2016 and the average at Harbin in 2015 and 2016. *Glyma.08G195300* and *Glyma.08G195400* were screened under Shenyang in 2015, Harbin in 2016, and the average at Harbin in 2015 and 2016. *Glyma.08G195500* and *Glyma.08G195700* were detected under Harbin in 2015, Harbin in 2016, and the average at Harbin in 2015 and 2016. *Glyma.08G195600* was scanned under Shenyang in 2015, the average at Shenyang in 2015 and 2016, Harbin in 2016, and the average at Harbin in 2015 and 2016. *Glyma.18G143700* was detected from Gm18:23052511 that overlapped the region of known loci, “SWPP 6-6” (Yao et al., 2015) and was detected under the condition of Shenyang in 2015, Shenyang in 2016, and the average at Shenyang in 2015 and 2016. These genes and beneficial haplotypes might be valuable for MAS in regulating SWPP of soybean.

## Discussion

Seed weight per plant, controlled by multiple genes, was an important component of seed yield in soybean. Thus far, some QTL have been identified based on linkage analysis from the SoyBase databank. However, few studies dissecting the genetic basis of SWPP in soybean via GWAS in combination with high-throughput SNPs and diverse accessions across multiple environments have been discussed, and even fewer candidate genes have been reported. In the present study, 185 soybean accessions were widely collected from China and used to conduct GWAS via high-throughput SNPs and diverse environments. In total, twenty SNPs were identified among twelve different soybean chromosomes, and these SNPs might have value for further breeding cultivars with appropriate SWPP.

In addition to the QTL associated with SWPP in the SoyBase databank, the genes associated with seed weight were referred for more accurately identifying the genomic region underlying the SWPP of soybean via the twenty SNPs used in the present study. Among the twenty SNPs, eight loci, including Gm02:27731352 located on Chr.2, Gm03:25789435 and Gm03:26388034 located on Chr.3, Gm12:24101071 located on Chr.12, Gm14:47326193 located on Chr.14, Gm15:45663581 located on Chr.15, and Gm19:19848537 and Gm19:19848932 located on Chr.19, were novel genes first reported in the present study. The genomic region of Chr.19, which included two close loci (Gm19:19848537 and Gm19:19848932), might be a primary region associated with SWPP. Additionally, another twelve SNPs were overlapped or located near known QTL (**Table 2**). Among these SNPs, one SNP (Gm18:23052511 located on Chr.18) was identified in the same region reported by Mian et al. (1996), and this polymorphism was also located near the locus named SWPP 6-6, which had been reported by Yao et al. (2015). A set of seven SNPs, including Gm02:17792241 on Chr.2, Gm08:15768591 and Gm08:15768603 on Chr.8, Gm10:34747895 and Gm10:34946492 on Chr.10, Gm12:31782089 on Chr.12, and Gm20:29716176 on Chr.20, were located in the same genomic region previously reported by Han et al. (2012) by using three RIL populations, and furthermore, among these seven SNPs, two close loci (Gm08:15768591 and Gm08:15768603 on Chr.8), Gm12:31782089 and Gm20:29716176 were also stably identified near previously reported loci (Sebolt et al., 2000; Zhang et al., 2004; Kato et al., 2014). Additionally, four genomic regions (Gm07:19488264 on Chr.7, Gm14:24306475 on Chr.14, Gm17:10196755 on Chr.17, and Gm19:11082560 on Chr.19) were also identified in previous studies (Csanadi et al., 2001; Hoeck et al., 2003; Teng et al., 2009). Moreover, some SNPs from candidate genes based on gene-based association analysis were found close to the loci that were verified in the present study by GWAS and previous studies. The main loci were Gm07:19488264, Gm08:15768591, Gm08:15768603, and Gm18:23052511. These four loci might be major genomic regions containing candidate genes associated with SWPP.

Currently, GWAS had been an effective method to acquire and confirm genes with a suitable LD block (Zhao et al., 2017). In the present study, a total of 99 candidate genes were identified in 200-kbp genomic regions based on the twenty association signals by GWAS with an LD block of approximately 214 kbp in length. Among these genes, those involved in hormone pathways, RNA regulation of transcription in plant development and maturation (*TTG2*, *AP2*, *MINI3*, and *C2H2*, for example), ubiquitin, transporting systems, and other metabolic processes associated with plant growth (ABC transporters and amino acid metabolism, for example), were considered as the important factors in determining the regulation of SWPP. Therefore, fourteen genes (*Glyma.02G159600*, *Glyma.02G173900*, *Glyma.07G157600*, *Glyma.07G157800*, *Glyma.07G157900*, *Glyma.08G195300*, *Glyma.08G195500*, *Glyma.08G195600*, *Glyma.08G195700*, *Glyma.08G195800*, *Glyma.17G127400*, *Glyma.17G127700*, *Glyma.19G057700*, and *Glyma.19G067900*) mainly belonging to transcription factor families of *AP2* and *C2H2* were proposed as responsible for the SWPP of soybean. Another five novel genes associated with the pathways of brassinosteroid, auxin, ethylene, and gibberellin were also regarded as the candidate genes, including *Glyma.10G129700*, *Glyma.10G130000*, *Glyma.15G240600*, *Glyma.17G127100*, and *Glyma.17G127500*. As E3 ubiquitin ligase genes catalyze the ubiquitination of proteins in regulating the growth of plants associated with SWPP (Xia et al., 2013; Yao et al., 2017), *Glyma.08G195200*, *Glyma.08G195400*, and *Glyma.08G195900*, belonging to the E3 ubiquitin family, might act as important factors in controlling SWPP. Additionally, in Arabidopsis, the regulators of amino acid metabolism and ABC transporters, which participate in auxin transport, played a key role in regulating plant growth and seed maturation (Wu et al., 2007; Less et al., 2010). Thus, *Glyma.02G159900*, *Glyma.08G196200*, *Glyma.12G163700*, *Glyma.12G163800*, *Glyma.14G207200*, *Glyma.14G207300*, *Glyma.14G207700*, *Glyma.14G207800*, *Glyma.17G127800*, and *Glyma.18G143700*, which belong to transport systems (ABC transporters, for example) and other main metabolic processes (amino acid metabolism, for example), might be novel genes associated with SWPP. To further accurately detect the candidate genes controlling SWPP, haplotype analysis of candidate genes was performed. As a result, nine genes and 15 beneficial haplotypes were detected by gene-based association analysis. Definitive function of all candidate genes would be discussed and verified in further studies.

## Author Contributions

YJ and XZ conceived the study and contributed to population development. JW contributed to phenotypic evaluation. WT and LQ contributed to genotyping. YH and WL contributed to the experimental design and drafting the manuscript. All authors contributed to and approved the final manuscript.

## Conflict of Interest Statement

The authors declare that the research was conducted in the absence of any commercial or financial relationships that could be construed as a potential conflict of interest.

## Footnotes

1 www.soybase.org

2 http://www.soybase.org

## Supplementary Material

FIGURE S1 Functional classifications of the candidate genes of seed weight per plant in soybean.

TABLE S1 List of 185 soybean accessions.

TABLE S2 Information of 33,149 SNPs.

TABLE S3 Haplotype analysis of candidate genes.

## Abbreviations

CMLM: compressed mixed linear model
GWAS: genome-wide association study
LD: linkage disequilibrium
MAF: minor allele frequency
PCA: principle component analysis
QTL: quantitative trait locus
SLAF-seq: specific-locus amplified fragment sequencing
SNPs: single-nucleotide polymorphisms
SWPP: seed weight per plant
