# Supplementary figures and images for: Identification of the Genomic Region Underlying Seed Weight per Plant in Soybean (Glycine max L. Merr.) via High-Throughput Single-Nucleotide Polymorphisms and a Genome-Wide Association Study
Source: Front Plant Sci. 2018 Oct 11;9:1392. doi: 10.3389/fpls.2018.01392 (PMC6194254; doi:10.3389/fpls.2018.01392)

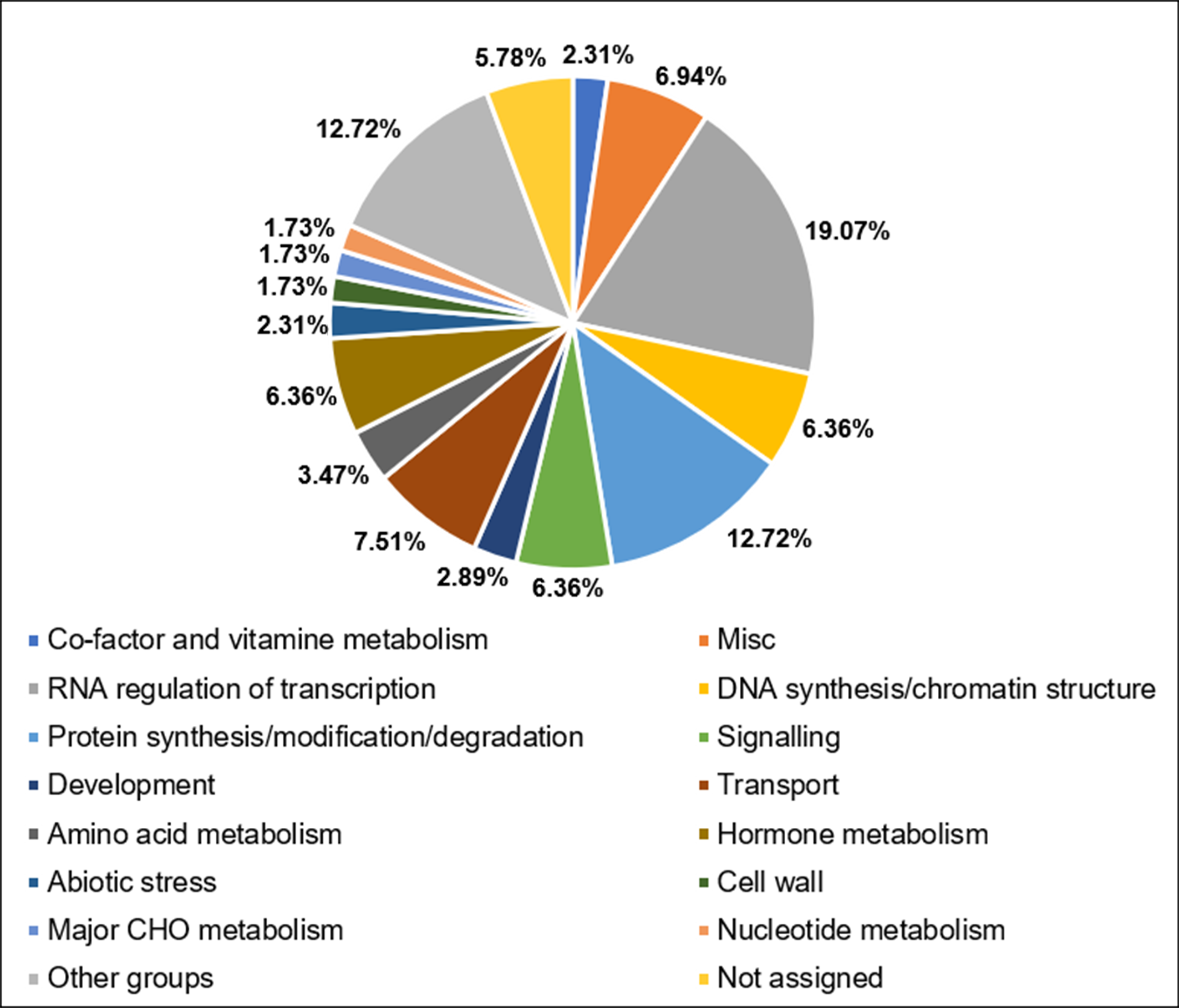

Supplement: FIGURE S1 — Functional classifications of the candidate genes of seed weight per plant in soybean. [file Image_1.TIF]
